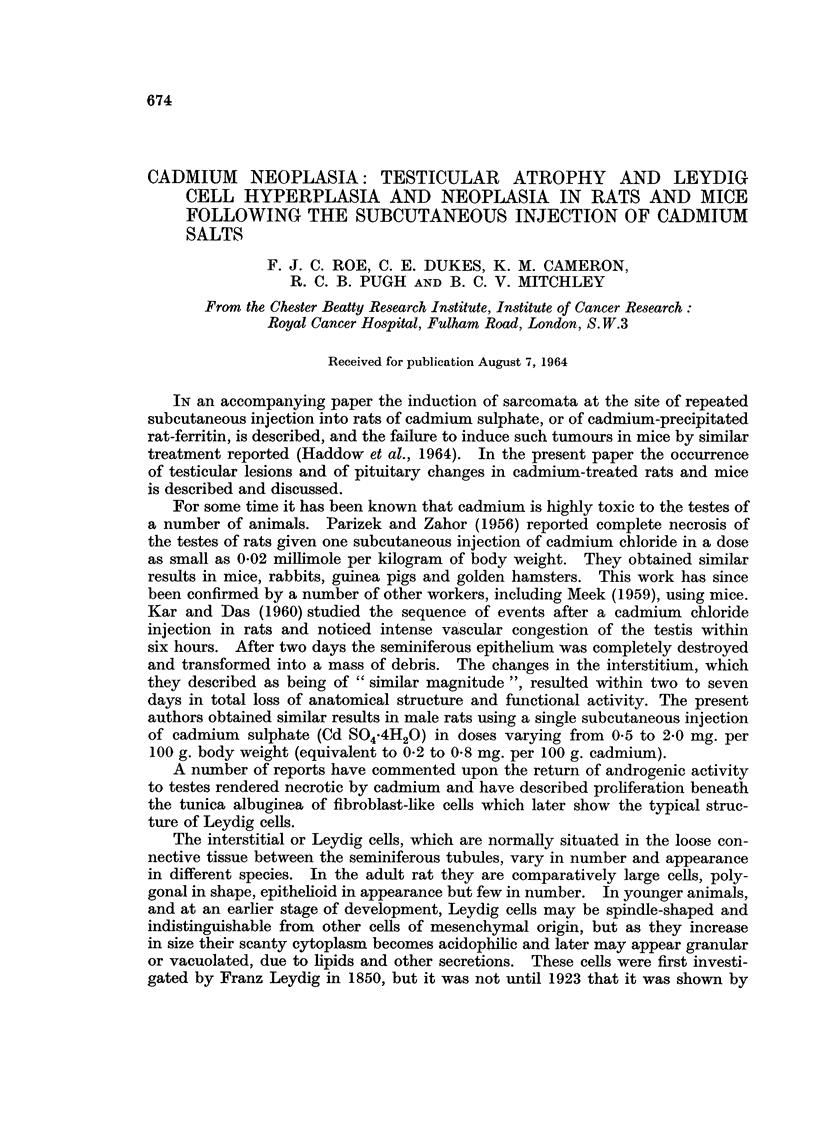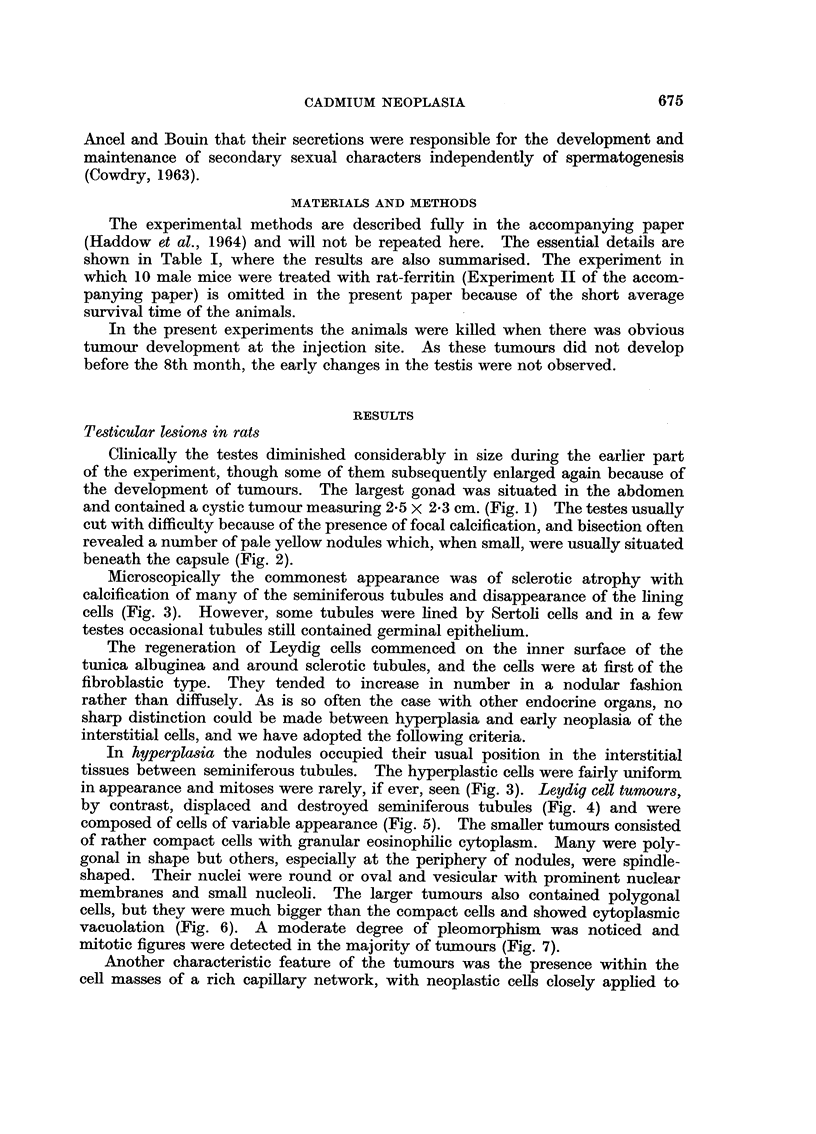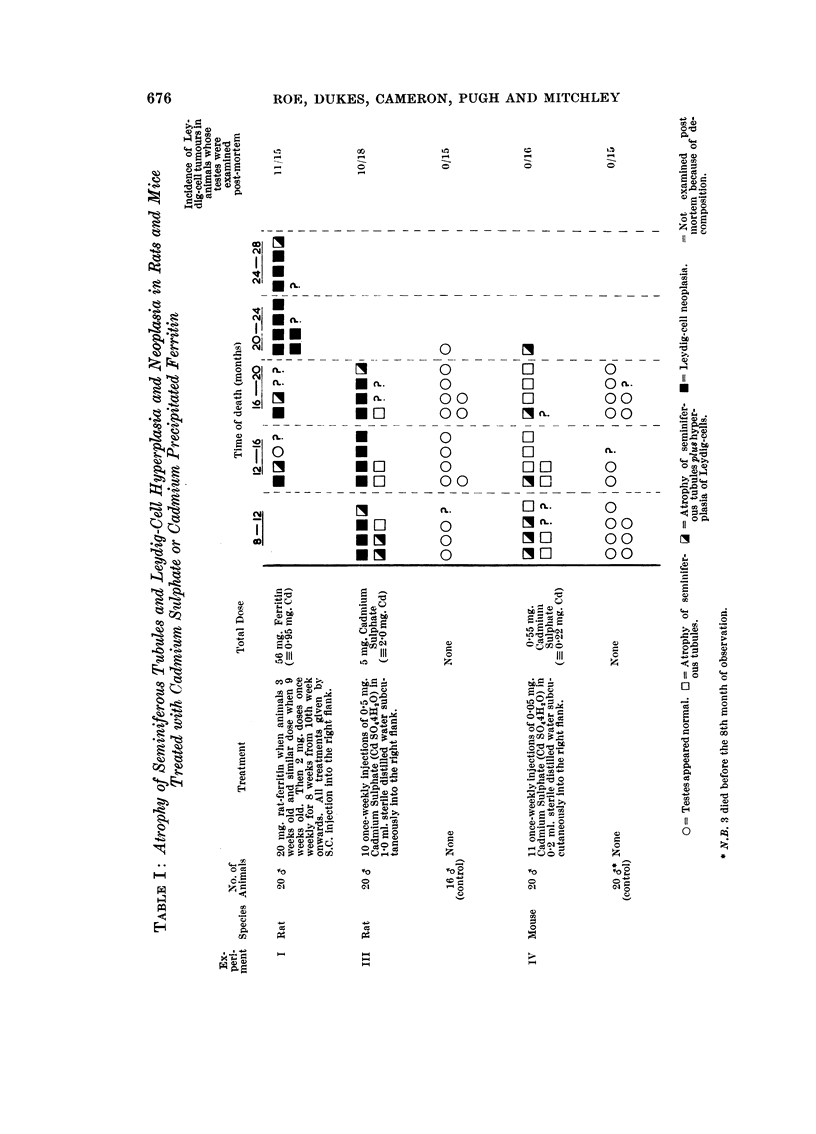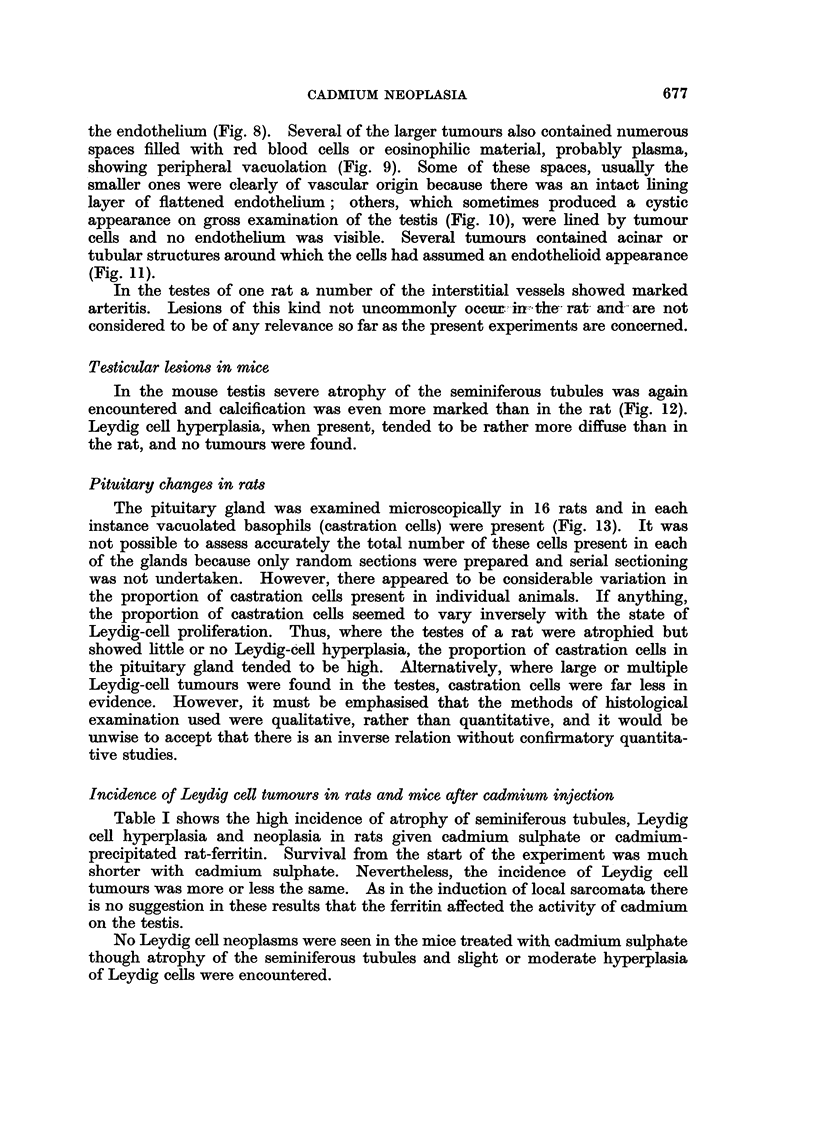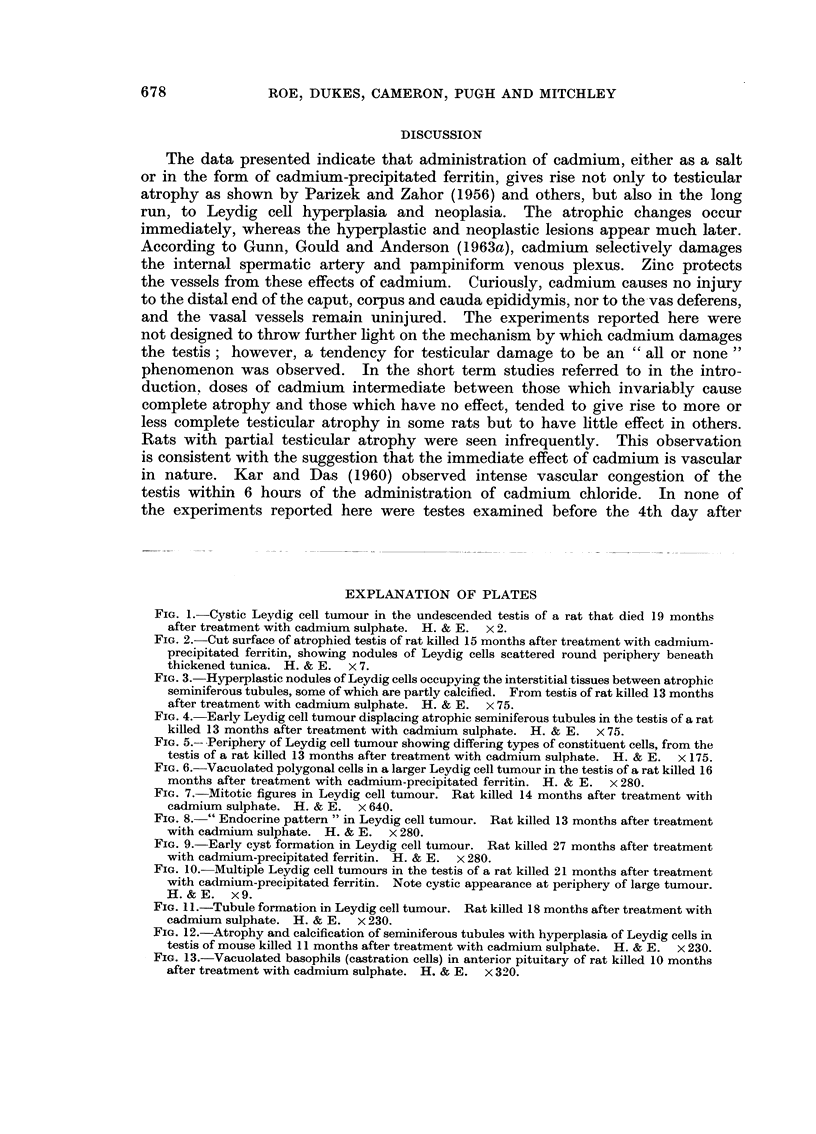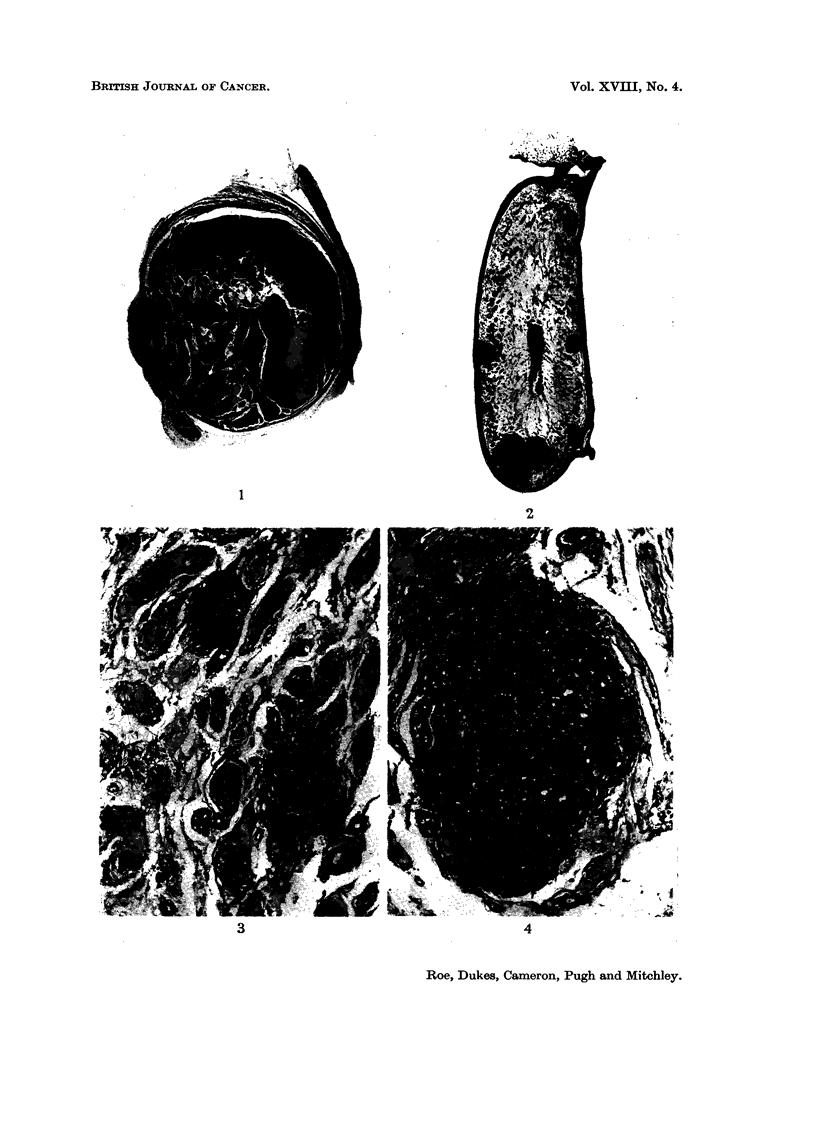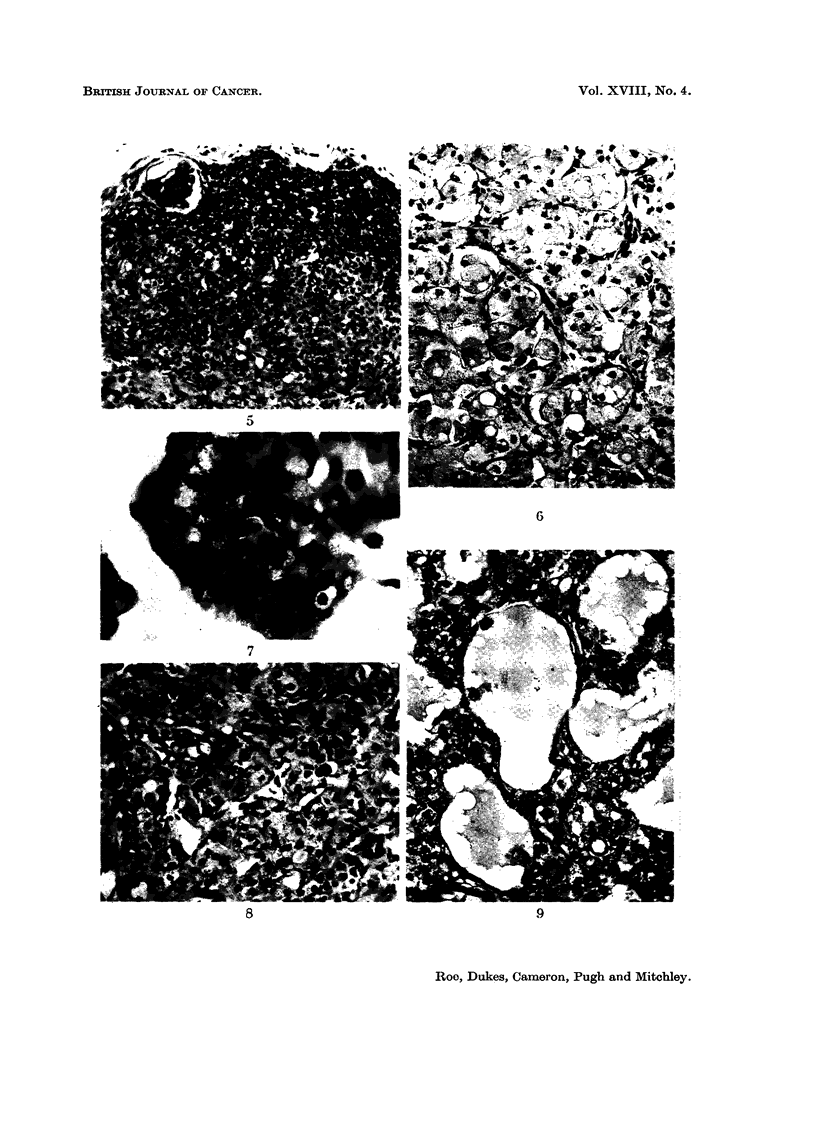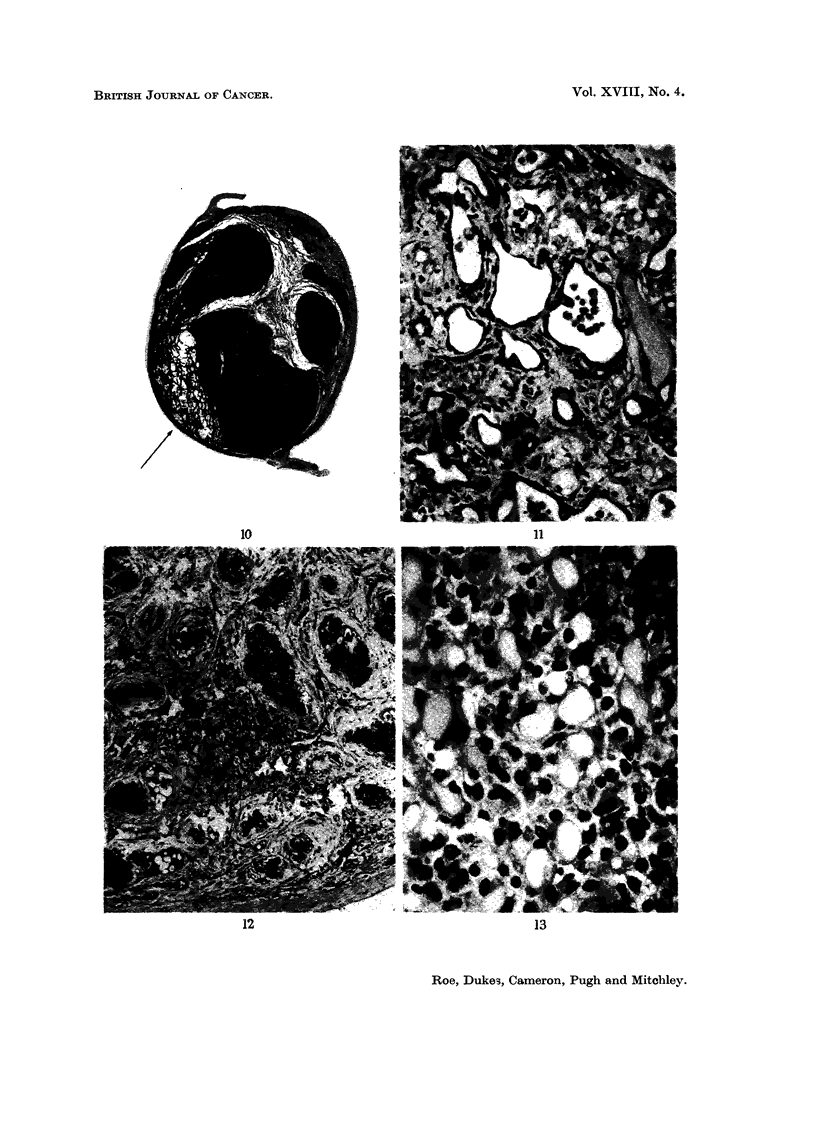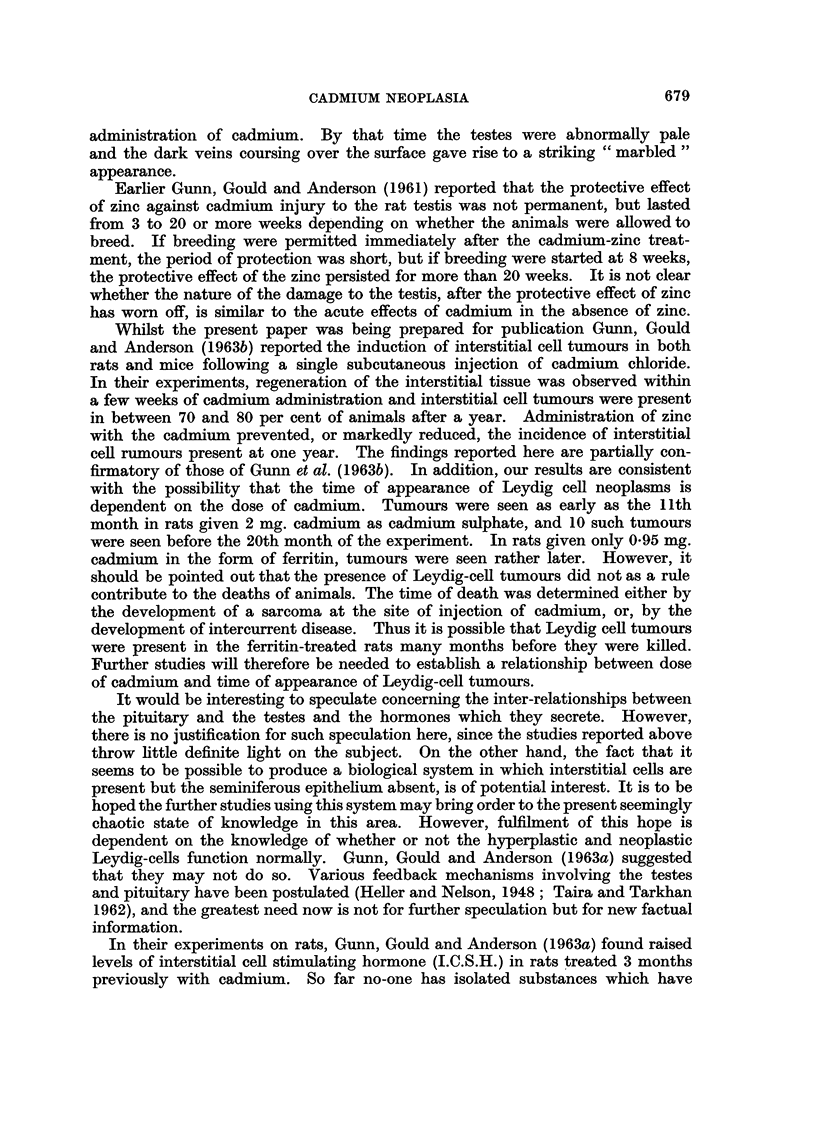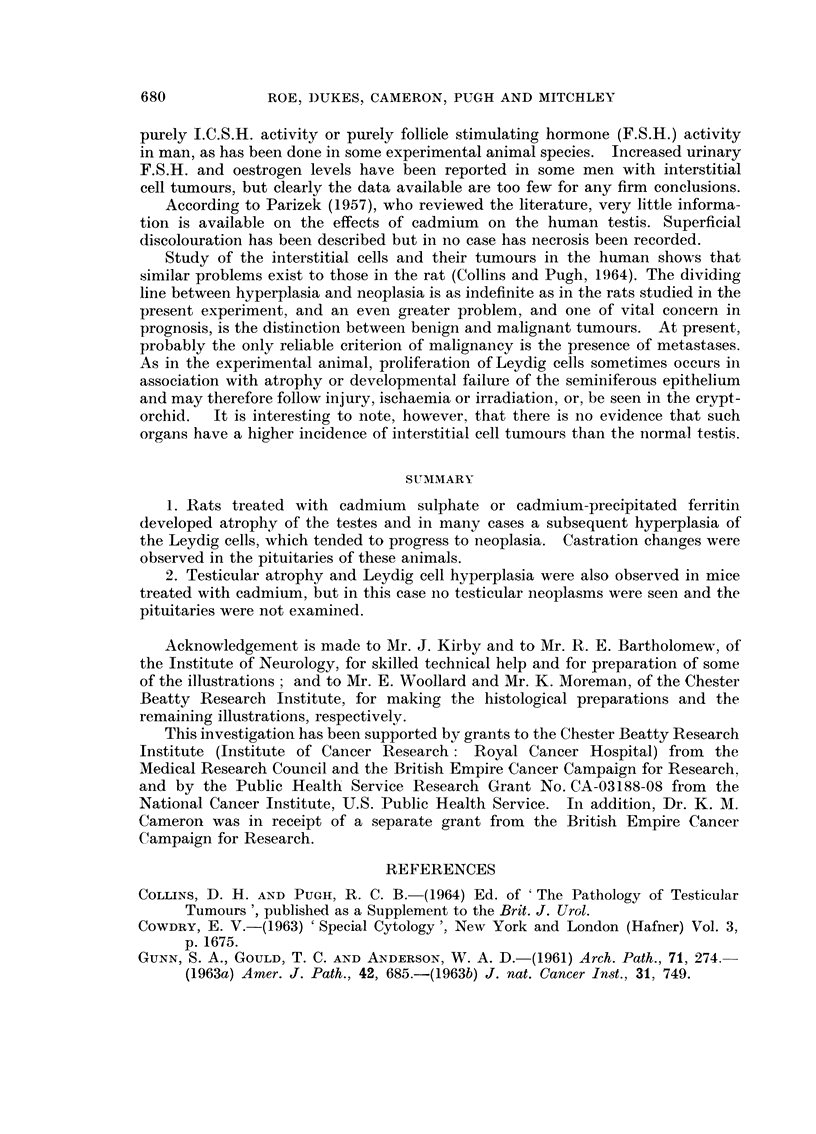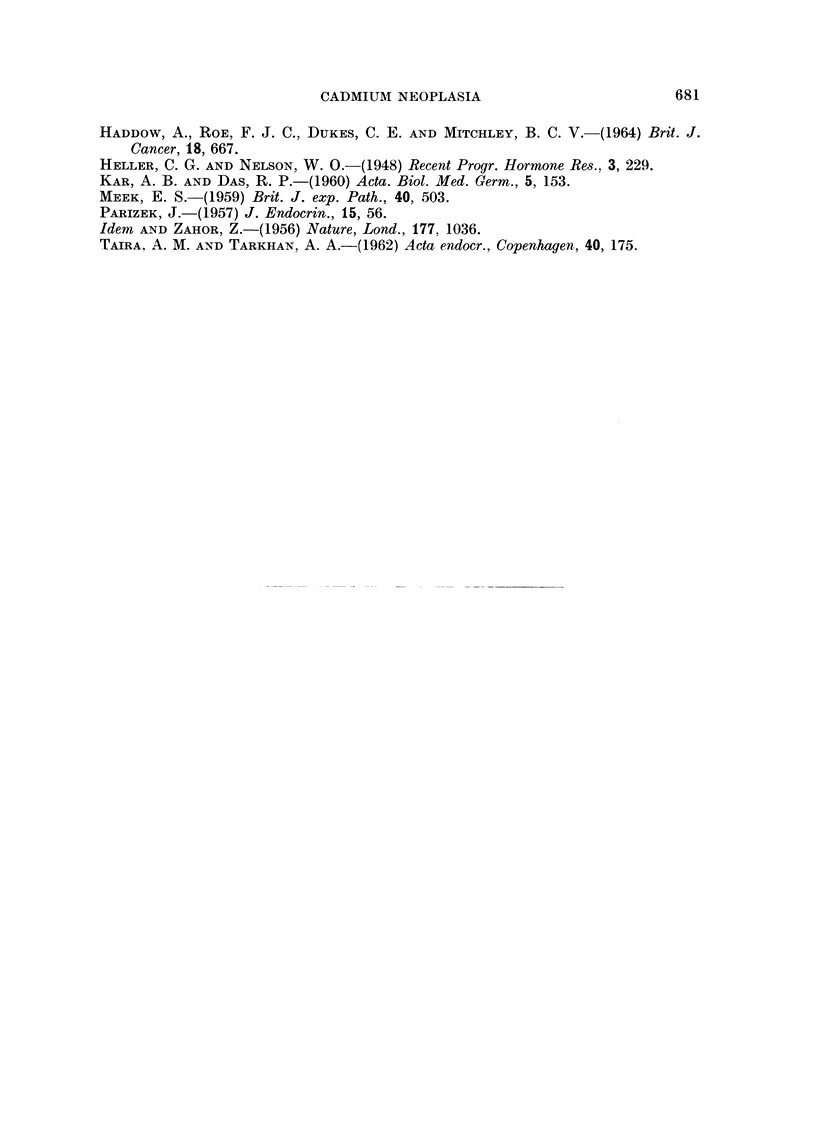# Cadmium Neoplasia: Testicular Atrophy and Leydig Cell Hyperplasia and Neoplasia in Rats and Mice Following the Subcutaneous Injection of Cadmium Salts

**DOI:** 10.1038/bjc.1964.77

**Published:** 1964-12

**Authors:** F. J. C. Roe, C. E. Dukes, K. M. Cameron, R. C. B. Pugh, B. C. V. Mitchley

## Abstract

**Images:**


### CADMIUM NEOPLASIA: TESTICULAR ATROPHY AND LEYDIG CELL HYPERPLASIA AND NEOPLASIA IN RATS AND MICE FOLLOWING THE SUBCUTANEOUS INJECTION OF CADMIUM SALTS

F. J. C. ROE, C. E. DUKES, K. M. CAMERON,
R. C. B. PUGH AND B. C. V. MITCHLEY

*From the Chester Beatty Research Institute, Institute of Cancer Research: Royal Cancer Hospital, Fulham Road, London, S.W.3*

Received for publication August 7, 1964

IN an accompanying paper the induction of sarcomata at the site of repeated subcutaneous injection into rats of cadmium sulphate, or of cadmium-precipitated rat-ferritin, is described, and the failure to induce such tumours in mice by similar treatment reported (Haddow *et al.*, 1964). In the present paper the occurrence of testicular lesions and of pituitary changes in cadmium-treated rats and mice is described and discussed.

For some time it has been known that cadmium is highly toxic to the testes of a number of animals. Parizek and Zahor (1956) reported complete necrosis of the testes of rats given one subcutaneous injection of cadmium chloride in a dose as small as 0·02 millimole per kilogram of body weight. They obtained similar results in mice, rabbits, guinea pigs and golden hamsters. This work has since been confirmed by a number of other workers, including Meek (1959), using mice. Kar and Das (1960) studied the sequence of events after a cadmium chloride injection in rats and noticed intense vascular congestion of the testis within six hours. After two days the seminiferous epithelium was completely destroyed and transformed into a mass of debris. The changes in the interstitium, which they described as being of " similar magnitude ", resulted within two to seven days in total loss of anatomical structure and functional activity. The present authors obtained similar results in male rats using a single subcutaneous injection of cadmium sulphate ($Cd\ SO_4{\cdot}4H_2O$) in doses varying from 0·5 to 2·0 mg. per 100 g. body weight (equivalent to 0·2 to 0·8 mg. per 100 g. cadmium).

A number of reports have commented upon the return of androgenic activity to testes rendered necrotic by cadmium and have described proliferation beneath the tunica albuginea of fibroblast-like cells which later show the typical structure of Leydig cells.

The interstitial or Leydig cells, which are normally situated in the loose connective tissue between the seminiferous tubules, vary in number and appearance in different species. In the adult rat they are comparatively large cells, polygonal in shape, epithelioid in appearance but few in number. In younger animals, and at an earlier stage of development, Leydig cells may be spindle-shaped and indistinguishable from other cells of mesenchymal origin, but as they increase in size their scanty cytoplasm becomes acidophilic and later may appear granular or vacuolated, due to lipids and other secretions. These cells were first investigated by Franz Leydig in 1850, but it was not until 1923 that it was shown by

Ancel and Bouin that their secretions were responsible for the development and maintenance of secondary sexual characters independently of spermatogenesis (Cowdry, 1963).

### MATERIALS AND METHODS

The experimental methods are described fully in the accompanying paper (Haddow *et al.*, 1964) and will not be repeated here. The essential details are shown in Table I, where the results are also summarised. The experiment in which 10 male mice were treated with rat-ferritin (Experiment II of the accompanying paper) is omitted in the present paper because of the short average survival time of the animals.

In the present experiments the animals were killed when there was obvious tumour development at the injection site. As these tumours did not develop before the 8th month, the early changes in the testis were not observed.

### RESULTS

### *Testicular lesions in rats*

Clinically the testes diminished considerably in size during the earlier part of the experiment, though some of them subsequently enlarged again because of the development of tumours. The largest gonad was situated in the abdomen and contained a cystic tumour measuring $2{\cdot}5 \times 2{\cdot}3$ cm. (Fig. 1) The testes usually cut with difficulty because of the presence of focal calcification, and bisection often revealed a number of pale yellow nodules which, when small, were usually situated beneath the capsule (Fig. 2).

Microscopically the commonest appearance was of sclerotic atrophy with calcification of many of the seminiferous tubules and disappearance of the lining cells (Fig. 3). However, some tubules were lined by Sertoli cells and in a few testes occasional tubules still contained germinal epithelium.

The regeneration of Leydig cells commenced on the inner surface of the tunica albuginea and around sclerotic tubules, and the cells were at first of the fibroblastic type. They tended to increase in number in a nodular fashion rather than diffusely. As is so often the case with other endocrine organs, no sharp distinction could be made between hyperplasia and early neoplasia of the interstitial cells, and we have adopted the following criteria.

In *hyperplasia* the nodules occupied their usual position in the interstitial tissues between seminiferous tubules. The hyperplastic cells were fairly uniform in appearance and mitoses were rarely, if ever, seen (Fig. 3). *Leydig cell tumours*, by contrast, displaced and destroyed seminiferous tubules (Fig. 4) and were composed of cells of variable appearance (Fig. 5). The smaller tumours consisted of rather compact cells with granular eosinophilic cytoplasm. Many were polygonal in shape but others, especially at the periphery of nodules, were spindle-shaped. Their nuclei were round or oval and vesicular with prominent nuclear membranes and small nucleoli. The larger tumours also contained polygonal cells, but they were much bigger than the compact cells and showed cytoplasmic vacuolation (Fig. 6). A moderate degree of pleomorphism was noticed and mitotic figures were detected in the majority of tumours (Fig. 7).

Another characteristic feature of the tumours was the presence within the cell masses of a rich capillary network, with neoplastic cells closely applied to

TABLE I: *Atrophy of Seminiferous Tubules and Leydig-Cell Hyperplasia and Neoplasia in Rats and Mice Treated with Cadmium Sulphate or Cadmium Precipitated Ferritin*

| Ex-periment | Species | No. of Animals | Treatment | Total Dose | Time of death (months) 8—12 | 12—16 | 16—20 | 20—24 | 24—28 | Incidence of Leydig-cell tumours in animals whose testes were examined post-mortem |
|---|---|---|---|---|---|---|---|---|---|---|
| I | Rat | 20 ♂ | 20 mg. rat-ferritin when animals 3 weeks old and similar dose when 9 weeks old. Then 2 mg. doses once weekly for 8 weeks from 10th week onwards. All treatments given by S.C. injection into the right flank. | 56 mg. Ferritin (≡0·95 mg. Cd) | | ■◪○? | ■◪?? | ■■■■ ■■? | ■■■◪ ? | 11/15 |
| III | Rat | 20 ♂ | 10 once-weekly injections of 0·5 mg. Cadmium Sulphate (Cd $SO_4 4H_2O$) in 1·0 ml. sterile distilled water subcutaneously into the right flank. | 5 mg. Cadmium Sulphate (≡2·0 mg. Cd) | ■■■◪ ◪◪□ | ■■■■ □□ | ■■■◪ □?? | | | 10/18 |
| | | 16 ♂ (control) | None | None | ○○○? | ○○○○ ○ | ○○○○ ○○ | ○ | | 0/15 |
| IV | Mouse | 20 ♂ | 11 once-weekly injections of 0·05 mg. Cadmium Sulphate (Cd $SO_4 4H_2O$) in 0·2 ml. sterile distilled water subcutaneously into the right flank. | 0·55 mg. Cadmium Sulphate (≡0·22 mg. Cd) | ◪◪◪□ □□?? | ◪□□□ □□ | ◪□□□ ? | ◪ | | 0/16 |
| | | 20 ♂* (control) | None | None | ○○○○ ○○○ | ○○? | ○○○○ ○○? | | | 0/15 |

○ = Testes appeared normal. □ = Atrophy of seminiferous tubules. ◪ = Atrophy of seminiferous tubules *plus* hyperplasia of Leydig-cells. ■ = Leydig-cell neoplasia. = Not examined post mortem because of decomposition.

* *N.B.* 3 died before the 8th month of observation.

the endothelium (Fig. 8). Several of the larger tumours also contained numerous spaces filled with red blood cells or eosinophilic material, probably plasma, showing peripheral vacuolation (Fig. 9). Some of these spaces, usually the smaller ones were clearly of vascular origin because there was an intact lining layer of flattened endothelium ; others, which sometimes produced a cystic appearance on gross examination of the testis (Fig. 10), were lined by tumour cells and no endothelium was visible. Several tumours contained acinar or tubular structures around which the cells had assumed an endothelioid appearance (Fig. 11).

In the testes of one rat a number of the interstitial vessels showed marked arteritis. Lesions of this kind not uncommonly occur in the rat and are not considered to be of any relevance so far as the present experiments are concerned.

*Testicular lesions in mice*

In the mouse testis severe atrophy of the seminiferous tubules was again encountered and calcification was even more marked than in the rat (Fig. 12). Leydig cell hyperplasia, when present, tended to be rather more diffuse than in the rat, and no tumours were found.

*Pituitary changes in rats*

The pituitary gland was examined microscopically in 16 rats and in each instance vacuolated basophils (castration cells) were present (Fig. 13). It was not possible to assess accurately the total number of these cells present in each of the glands because only random sections were prepared and serial sectioning was not undertaken. However, there appeared to be considerable variation in the proportion of castration cells present in individual animals. If anything, the proportion of castration cells seemed to vary inversely with the state of Leydig-cell proliferation. Thus, where the testes of a rat were atrophied but showed little or no Leydig-cell hyperplasia, the proportion of castration cells in the pituitary gland tended to be high. Alternatively, where large or multiple Leydig-cell tumours were found in the testes, castration cells were far less in evidence. However, it must be emphasised that the methods of histological examination used were qualitative, rather than quantitative, and it would be unwise to accept that there is an inverse relation without confirmatory quantitative studies.

*Incidence of Leydig cell tumours in rats and mice after cadmium injection*

Table I shows the high incidence of atrophy of seminiferous tubules, Leydig cell hyperplasia and neoplasia in rats given cadmium sulphate or cadmium-precipitated rat-ferritin. Survival from the start of the experiment was much shorter with cadmium sulphate. Nevertheless, the incidence of Leydig cell tumours was more or less the same. As in the induction of local sarcomata there is no suggestion in these results that the ferritin affected the activity of cadmium on the testis.

No Leydig cell neoplasms were seen in the mice treated with cadmium sulphate though atrophy of the seminiferous tubules and slight or moderate hyperplasia of Leydig cells were encountered.

### DISCUSSION

The data presented indicate that administration of cadmium, either as a salt or in the form of cadmium-precipitated ferritin, gives rise not only to testicular atrophy as shown by Parizek and Zahor (1956) and others, but also in the long run, to Leydig cell hyperplasia and neoplasia. The atrophic changes occur immediately, whereas the hyperplastic and neoplastic lesions appear much later. According to Gunn, Gould and Anderson (1963*a*), cadmium selectively damages the internal spermatic artery and pampiniform venous plexus. Zinc protects the vessels from these effects of cadmium. Curiously, cadmium causes no injury to the distal end of the caput, corpus and cauda epididymis, nor to the vas deferens, and the vasal vessels remain uninjured. The experiments reported here were not designed to throw further light on the mechanism by which cadmium damages the testis ; however, a tendency for testicular damage to be an " all or none " phenomenon was observed. In the short term studies referred to in the introduction, doses of cadmium intermediate between those which invariably cause complete atrophy and those which have no effect, tended to give rise to more or less complete testicular atrophy in some rats but to have little effect in others. Rats with partial testicular atrophy were seen infrequently. This observation is consistent with the suggestion that the immediate effect of cadmium is vascular in nature. Kar and Das (1960) observed intense vascular congestion of the testis within 6 hours of the administration of cadmium chloride. In none of the experiments reported here were testes examined before the 4th day after

### EXPLANATION OF PLATES

FIG. 1.—Cystic Leydig cell tumour in the undescended testis of a rat that died 19 months after treatment with cadmium sulphate. H. & E. ×2.

FIG. 2.—Cut surface of atrophied testis of rat killed 15 months after treatment with cadmium-precipitated ferritin, showing nodules of Leydig cells scattered round periphery beneath thickened tunica. H. & E. ×7.

FIG. 3.—Hyperplastic nodules of Leydig cells occupying the interstitial tissues between atrophic seminiferous tubules, some of which are partly calcified. From testis of rat killed 13 months after treatment with cadmium sulphate. H. & E. ×75.

FIG. 4.—Early Leydig cell tumour displacing atrophic seminiferous tubules in the testis of a rat killed 13 months after treatment with cadmium sulphate. H. & E. ×75.

FIG. 5.—Periphery of Leydig cell tumour showing differing types of constituent cells, from the testis of a rat killed 13 months after treatment with cadmium sulphate. H. & E. ×175.

FIG. 6.—Vacuolated polygonal cells in a larger Leydig cell tumour in the testis of a rat killed 16 months after treatment with cadmium-precipitated ferritin. H. & E. ×280.

FIG. 7.—Mitotic figures in Leydig cell tumour. Rat killed 14 months after treatment with cadmium sulphate. H. & E. ×640.

FIG. 8.—" Endocrine pattern " in Leydig cell tumour. Rat killed 13 months after treatment with cadmium sulphate. H. & E. ×280.

FIG. 9.—Early cyst formation in Leydig cell tumour. Rat killed 27 months after treatment with cadmium-precipitated ferritin. H. & E. ×280.

FIG. 10.—Multiple Leydig cell tumours in the testis of a rat killed 21 months after treatment with cadmium-precipitated ferritin. Note cystic appearance at periphery of large tumour. H. & E. ×9.

FIG. 11.—Tubule formation in Leydig cell tumour. Rat killed 18 months after treatment with cadmium sulphate. H. & E. ×230.

FIG. 12.—Atrophy and calcification of seminiferous tubules with hyperplasia of Leydig cells in testis of mouse killed 11 months after treatment with cadmium sulphate. H. & E. ×230.

FIG. 13.—Vacuolated basophils (castration cells) in anterior pituitary of rat killed 10 months after treatment with cadmium sulphate. H. & E. ×320.

1

2

3

4

Roe, Dukes, Cameron, Pugh and Mitchley.

5

6

7

8

9

10

11

12

13

administration of cadmium. By that time the testes were abnormally pale and the dark veins coursing over the surface gave rise to a striking " marbled " appearance.

Earlier Gunn, Gould and Anderson (1961) reported that the protective effect of zinc against cadmium injury to the rat testis was not permanent, but lasted from 3 to 20 or more weeks depending on whether the animals were allowed to breed. If breeding were permitted immediately after the cadmium-zinc treatment, the period of protection was short, but if breeding were started at 8 weeks, the protective effect of the zinc persisted for more than 20 weeks. It is not clear whether the nature of the damage to the testis, after the protective effect of zinc has worn off, is similar to the acute effects of cadmium in the absence of zinc.

Whilst the present paper was being prepared for publication Gunn, Gould and Anderson (1963*b*) reported the induction of interstitial cell tumours in both rats and mice following a single subcutaneous injection of cadmium chloride. In their experiments, regeneration of the interstitial tissue was observed within a few weeks of cadmium administration and interstitial cell tumours were present in between 70 and 80 per cent of animals after a year. Administration of zinc with the cadmium prevented, or markedly reduced, the incidence of interstitial cell rumours present at one year. The findings reported here are partially confirmatory of those of Gunn *et al.* (1963*b*). In addition, our results are consistent with the possibility that the time of appearance of Leydig cell neoplasms is dependent on the dose of cadmium. Tumours were seen as early as the 11th month in rats given 2 mg. cadmium as cadmium sulphate, and 10 such tumours were seen before the 20th month of the experiment. In rats given only 0·95 mg. cadmium in the form of ferritin, tumours were seen rather later. However, it should be pointed out that the presence of Leydig-cell tumours did not as a rule contribute to the deaths of animals. The time of death was determined either by the development of a sarcoma at the site of injection of cadmium, or, by the development of intercurrent disease. Thus it is possible that Leydig cell tumours were present in the ferritin-treated rats many months before they were killed. Further studies will therefore be needed to establish a relationship between dose of cadmium and time of appearance of Leydig-cell tumours.

It would be interesting to speculate concerning the inter-relationships between the pituitary and the testes and the hormones which they secrete. However, there is no justification for such speculation here, since the studies reported above throw little definite light on the subject. On the other hand, the fact that it seems to be possible to produce a biological system in which interstitial cells are present but the seminiferous epithelium absent, is of potential interest. It is to be hoped the further studies using this system may bring order to the present seemingly chaotic state of knowledge in this area. However, fulfilment of this hope is dependent on the knowledge of whether or not the hyperplastic and neoplastic Leydig-cells function normally. Gunn, Gould and Anderson (1963*a*) suggested that they may not do so. Various feedback mechanisms involving the testes and pituitary have been postulated (Heller and Nelson, 1948 ; Taira and Tarkhan 1962), and the greatest need now is not for further speculation but for new factual information.

In their experiments on rats, Gunn, Gould and Anderson (1963*a*) found raised levels of interstitial cell stimulating hormone (I.C.S.H.) in rats treated 3 months previously with cadmium. So far no-one has isolated substances which have purely I.C.S.H. activity or purely follicle stimulating hormone (F.S.H.) activity in man, as has been done in some experimental animal species. Increased urinary F.S.H. and oestrogen levels have been reported in some men with interstitial cell tumours, but clearly the data available are too few for any firm conclusions.

According to Parizek (1957), who reviewed the literature, very little information is available on the effects of cadmium on the human testis. Superficial discolouration has been described but in no case has necrosis been recorded.

Study of the interstitial cells and their tumours in the human shows that similar problems exist to those in the rat (Collins and Pugh, 1964). The dividing line between hyperplasia and neoplasia is as indefinite as in the rats studied in the present experiment, and an even greater problem, and one of vital concern in prognosis, is the distinction between benign and malignant tumours. At present, probably the only reliable criterion of malignancy is the presence of metastases. As in the experimental animal, proliferation of Leydig cells sometimes occurs in association with atrophy or developmental failure of the seminiferous epithelium and may therefore follow injury, ischaemia or irradiation, or, be seen in the cryptorchid. It is interesting to note, however, that there is no evidence that such organs have a higher incidence of interstitial cell tumours than the normal testis.

### SUMMARY

1. Rats treated with cadmium sulphate or cadmium-precipitated ferritin developed atrophy of the testes and in many cases a subsequent hyperplasia of the Leydig cells, which tended to progress to neoplasia. Castration changes were observed in the pituitaries of these animals.

2. Testicular atrophy and Leydig cell hyperplasia were also observed in mice treated with cadmium, but in this case no testicular neoplasms were seen and the pituitaries were not examined.

Acknowledgement is made to Mr. J. Kirby and to Mr. R. E. Bartholomew, of the Institute of Neurology, for skilled technical help and for preparation of some of the illustrations ; and to Mr. E. Woollard and Mr. K. Moreman, of the Chester Beatty Research Institute, for making the histological preparations and the remaining illustrations, respectively.

This investigation has been supported by grants to the Chester Beatty Research Institute (Institute of Cancer Research : Royal Cancer Hospital) from the Medical Research Council and the British Empire Cancer Campaign for Research, and by the Public Health Service Research Grant No. CA-03188-08 from the National Cancer Institute, U.S. Public Health Service. In addition, Dr. K. M. Cameron was in receipt of a separate grant from the British Empire Cancer Campaign for Research.

### REFERENCES

COLLINS, D. H. AND PUGH, R. C. B.—(1964) Ed. of ' The Pathology of Testicular Tumours ', published as a Supplement to the *Brit. J. Urol.*

COWDRY, E. V.—(1963) ' Special Cytology ', New York and London (Hafner) Vol. 3, p. 1675.

GUNN, S. A., GOULD, T. C. AND ANDERSON, W. A. D.—(1961) *Arch. Path.*, **71**, 274.—(1963*a*) *Amer. J. Path.*, **42**, 685.—(1963*b*) *J. nat. Cancer Inst.*, **31**, 749.

HADDOW, A., ROE, F. J. C., DUKES, C. E. AND MITCHLEY, B. C. V.—(1964) *Brit. J. Cancer*, **18**, 667.
HELLER, C. G. AND NELSON, W. O.—(1948) *Recent Progr. Hormone Res.*, **3**, 229.
KAR, A. B. AND DAS, R. P.—(1960) *Acta. Biol. Med. Germ.*, **5**, 153.
MEEK, E. S.—(1959) *Brit. J. exp. Path.*, **40**, 503.
PARIZEK, J.—(1957) *J. Endocrin.*, **15**, 56.
*Idem* AND ZAHOR, Z.—(1956) *Nature, Lond.*, **177**, 1036.
TAIRA, A. M. AND TARKHAN, A. A.—(1962) *Acta endocr., Copenhagen*, **40**, 175.